# Research and implementation of intelligent clothing personalized customization system based on deep learning

**DOI:** 10.1038/s41598-026-40436-3

**Published:** 2026-03-04

**Authors:** Yeyue Lu

**Affiliations:** Fashion Design, University of Southampton, Park Ave, Winchester, SO23 8DL UK

**Keywords:** Multimodal deep learning, Anthropometric extraction, Garment simulation, Style preference modeling, Microservices architecture, Engineering, Mathematics and computing

## Abstract

This study presents an intelligent personalized garment customization system that integrates deep learning methodologies. The system employs a microservices architecture to unify four core modules: body measurement data extraction, style preference learning, virtual try-on visualization, and design recommendation generation. We propose a novel CNN-Transformer-GAN architecture, specifically tailored for personalized garment design tasks, achieving exceptional accuracy. Experimental results demonstrate that the system attains a mean absolute error (MAE) of 0.38 cm in body measurement, an accuracy of 87.4% in style matching, and a response time of 285 ms. Compared to existing approaches, the proposed system improves measurement accuracy by 38.7% and delivers visualization quality comparable to metaverse-based systems. To evaluate user experience, we conducted two complementary studies: (1) a controlled single-blind user study with 120 participants, which yielded satisfaction scores between 4.42 and 4.65 across recommendation accuracy, interface usability, and visualization quality; and (2) a large-scale deployment test involving 250 users, which reported an average overall satisfaction score of 4.55 out of 5.0. This research helps bridge the gap between artificial intelligence and personalized fashion design, advancing resource-efficient customization and better alignment with consumer needs in the apparel industry. By integrating state-of-the-art deep learning techniques with responsive user preference modeling, the system offers an innovative solution for intelligent garment customization.

## Introduction

### Research background and significance

The industry of clothing personalization has undergone significant transformation driven by advances in digital technology and evolving consumer behaviors. The shift away from the “one-size-fits-all” model particularly resonates with image-conscious consumers who increasingly demand garments tailored to their individual tastes, lifestyles, and precise body dimensions^1^. Traditional bespoke systems, however, face persistent challenges—including inefficient design workflows, measurement inaccuracies, limited fashion variety, and a misalignment between final products and actual consumer preferences^2^—which hinder the scalable delivery of truly personalized apparel.

Deep learning technologies offer promising solutions to these complex issues by effectively analyzing intricate visual and behavioral patterns, recognizing latent user preferences, and even generating novel design alternatives^3^. Wang et al.^4^ highlight how intelligent techniques enhance interactive personalized garment design systems, enabling more intuitive and fashion-responsive user experiences. Deep learning further facilitates the implementation of advanced functionalities—such as webcam-based virtual try-on, image-driven body measurement extraction, and automated pattern generation^5^—thereby significantly narrowing the gap between design conception and physical realization.

Critically, this technological integration also contributes to sustainability in fashion. By enabling precise, on-demand production aligned with individual demand, personalized systems reduce overproduction and inventory surplus—key drivers of textile waste. According to recent lifecycle assessments, AI-driven customization can lower material waste by up to 30% compared to mass production^6^. Furthermore, better-fitting and stylistically relevant garments increase wear frequency and product lifespan, indirectly supporting circular fashion principles^7^. These efficiencies not only enhance consumer satisfaction through more “poignant” (i.e., emotionally and functionally resonant) clothing^2^, but also enable innovative business models that jointly optimize personalization and sustainable manufacturing^8^.

### Research status

Research into intelligent clothing personalization systems has been on the rise recently. Sulthana^6^ examines fashion recommender systems based on deep learning, noting the evolution from traditional feature engineering to advanced neural styled attributes neural networks. Da’u and Salim^7^ discuss the innovative approaches in recommendation systems, particularly focusing on the categorization of deep learning techniques and emphasizing the role of CNNs and GANs in fashion image feature extraction.

In applications, Shi et al.^8^ present advances in personalized modeling for ethnic clothing customization, demonstrating preservation of cultural elements while incorporating modern capabilities. Sun^9^ details virtual intelligent 3D systems for clothing structure design, illustrating the feasibility of transitioning from 2D patternmaking to interactive 3D environments.

Despite these advances, significant gaps persist. Akram et al.^10^ identify challenges in data integration across design, manufacturing, and retail stages in Fashion Industry 4.0. Papachristou et al.^11^ demonstrate through case studies that implementation of machine learning in clothing manufacture faces organizational and technological barriers. These gaps present opportunities for developing comprehensive frameworks that connect consumer preferences, design processes, and manufacturing capabilities through unified deep learning architectures.

**Table 1:** Comparison of existing approaches and the proposed System.

Feature	Manual input/size charts	3D Scan-Based Tailoring (e.g^13^.,)	Modular AI Manufacturing (e.g^14^.,)	Ours
Relational Database	Rule-based or collaborative filtering	3D body scanning	Limited anthropometric integration	Image-based deep learning estimation (MAE: 0.38 cm)
Vector Database	Static overlays	Not addressed	Basic user profiles	Multimodal CNN-Transformer embedding with adaptive learning
Document Store	Item-level similarity	Physics-aware but offline	Not implemented	Real-time GAN-based virtual try-on with fabric simulation

### Research objectives and content

This research aims to develop an intelligent clothing personalized customization system leveraging deep learning to bridge the gap between consumer preferences and garment production. The focus of the project is to develop an all-in-one solution that automatically captures body dimensions, learns style preferences, allows for virtual try-ons, and recommends designs. Following Ye and Su^12^, who described a custom-made metaverse twin within the metaverse, we envisioned an elaborate customization approach and its operationalization within retail stores.

The system is composed of four parts: an image-based anthropometric feature extraction body measurement algorithm, a user profile adaptive style preference learning model, a virtual reality system for realistic visualizations, and a design personalization recommendation system. While Wang et al.^13^ showed how machine learning enhances a process called 3D reverse design, our work integrates multimodal data and more advanced deep learning within a modular system proposed by Wan et al.^14^, which was focused on modular systems in AI-driven manufacturing, as shown in Table 1.

As with any research, some boundaries are set. The primary focus has been on ready-to-wear and tailored garments, but specialized clothing was not included. The diversity of the training data, as Yu and Zhao^15^ comment on, can certainly have an impact across populations. Additionally, computational resources for real-time rendering present implementation challenges. Despite these limitations, the system architecture integrates recent deep learning advances with practical user experience considerations, positioning this research to make significant contributions to intelligent clothing customization.

### Technical route and research methods

This research adopts a systematic methodology combining design science principles with experimental evaluations. Drawing from Han’s^16^ work on AI-driven pattern making, we implement a multi-stage process including requirement analysis, algorithm design, prototype implementation, and system evaluation, allowing continuous refinement through feedback loops.

**Fig. 1:**
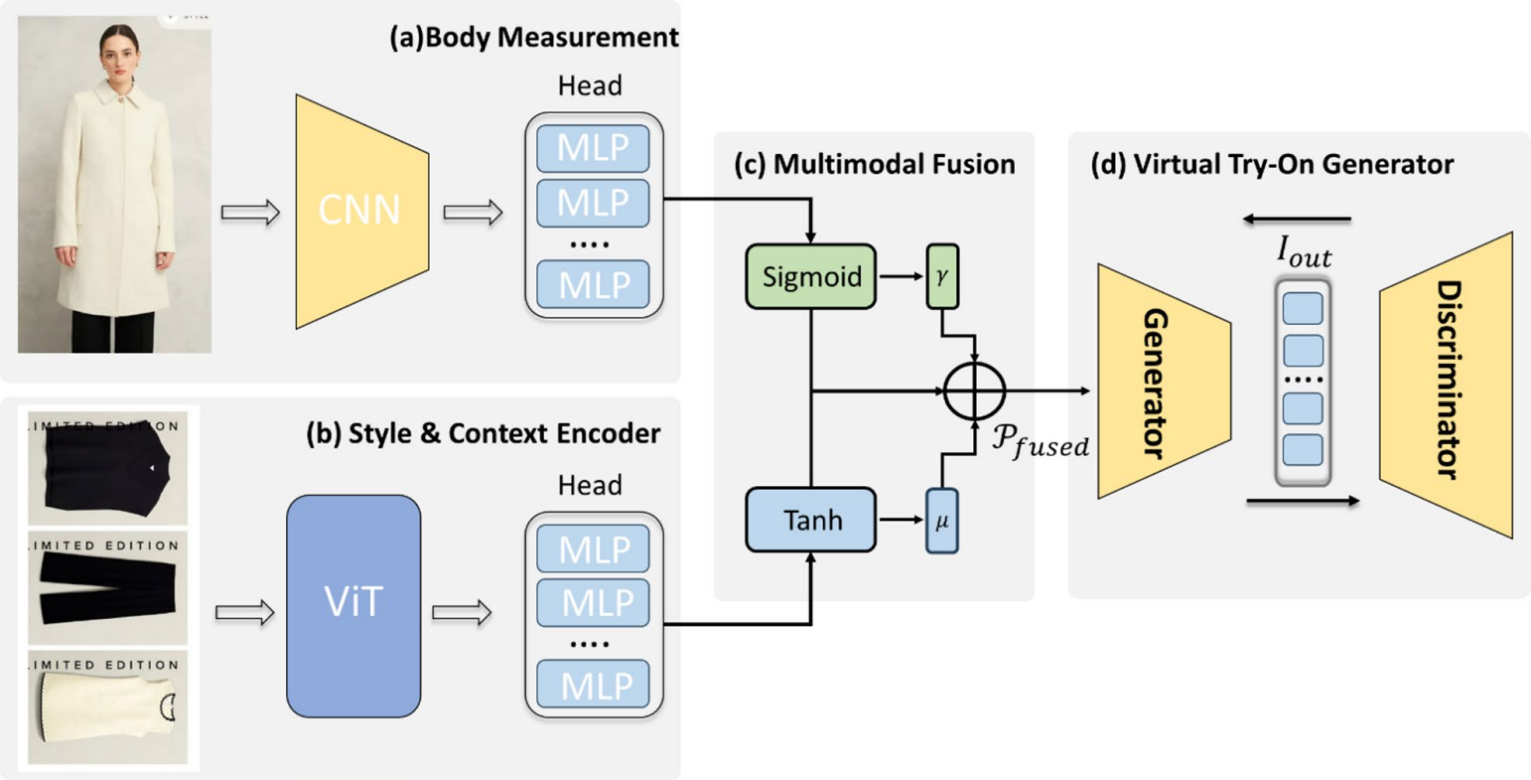
Overview of Multimodal Transformer-CNN-GAN framework.

The technical framework incorporates a multi-layered architecture (Fig. 1) with data acquisition, feature extraction, preference modeling, and recommendation generation layers. For data acquisition, we employ multimodal sources including 2D images, 3D scans, and user interaction data. The feature extraction layer utilizes CNNs for image processing and transformer-based models for style analysis, following Butteddi and Butteddi’s^17^ generative AI approach. Building upon Wu et al.‘s^18^ anthropometric processing techniques, our system incorporates enhanced body measurement algorithms combining computer vision with deep learning.

## Theoretical foundation and key technologies

### Deep learning fundamentals

Deep learning has been widely adopted for garment image analysis, style modeling, and design generation. CNNs remain dominant for garment recognition due to their hierarchical feature extraction capabilities^7^, while GANs enable market-aware creative design through adversarial generation and evaluation^21^. Recent efforts have explored hybrid CNN–Transformer^24,25^ architectures to jointly capture local details and global semantics. Additionally, transfer learning and knowledge distillation have alleviated data scarcity and enabled efficient deployment on resource-constrained devices^2,22^.

However, existing approaches typically address isolated tasks (e.g., recognition or generation) and lack a unified, end-to-end multimodal framework for personalized customization. Moreover, most systems treat modules independently, without formalizing cross-modal fusion mechanisms, limiting information synergy.

In this work, we propose a tightly integrated CNN–Transformer–GAN architecture with a learnable multimodal fusion strategy to jointly optimize body measurement, preference modeling, and design generation.

### Computer vision for garment analysis

Semantic segmentation (e.g., U-Net-based) now achieves pixel-level garment parsing^9^, and multi-scale CNNs effectively encode texture, color, and shape across abstraction levels^23^. Multimodal frameworks combining images and text further improve recognition robustness in ambiguous scenarios^5^. Interactive style transfer methods also enable user-in-the-loop design^19^.

Yet, current 2D-based methods struggle to accurately reconstruct 3D garment structure and material behavior under real-world conditions, and few systems dynamically incorporate user feedback into the generation loop.

We address this by fusing sparse anthropometric cues with high-dimensional style representations to build a context-aware recommendation and rendering pipeline, enhanced with an interactive refinement mechanism.

### Human body measurement and modeling

3D body scanning is the gold standard for high-fidelity anthropometry, enabling pose-adaptive modeling and automated pattern generation^13^. Alternatively, 2D vision-based methods using front/side photos offer greater accessibility through contour-based and proportion-constrained estimation^18^. Nevertheless, 2D approaches suffer from occlusion and clothing deformation, while 3D solutions remain costly and impractical for mass adoption. Crucially, few systems model the propagation of measurement uncertainty into downstream design or virtual try-on stages, compromising overall consistency.

Our approach employs a lightweight cnn to improve robustness from limited image inputs and explicitly incorporates measurement confidence into the virtual try-on and recommendation modules, ensuring end-to-end coherence.

### Recommendation systems in fashion

Recent advances in fashion recommendation systems integrate both user body characteristics and style preferences to deliver personalized suggestions. Hybrid models combining collaborative filtering with content-based methods have shown superior performance by leveraging both behavioral data and item features, effectively balancing personal taste with novelty^6^. Multi-modal approaches further enhance preference modeling by fusing visual, textual, and interaction signals. For instance, Yang^3^combined decision trees with deep representation learning for interpretable style recommendations, while Kachbal et al.^9^ highlighted the shift from static features to dynamic preference modeling via attention mechanisms that adapt feature importance based on user behavior.

Context-awareness has also become central: systems now incorporate situational factors such as occasion, season, and location^18^, and increasingly account for social and cultural dimensions in clothing choices^24^. Interactive frameworks, like the one proposed by Wang et al.^4^, allow users to refine design preferences in real time, reflecting a move toward co-creative customization.

However, most existing fashion recommenders treat body measurements and style preferences as independent inputs, without modeling their interaction—e.g., how a user’s body shape influences their acceptance of certain silhouettes or cuts. Moreover, contextual factors are often handcrafted or loosely coupled with deep representations, limiting adaptability. Crucially, few systems close the loop between recommendation, virtual try-on, and user feedback in a unified pipeline.

To the best of our knowledge, no prior work has explicitly learned a joint embedding of anthropometric and visual style features in a shared latent space for personalized fashion recommendation. Existing approaches either rely on separate pipelines for size prediction and style suggestion^18,22^, or incorporate body shape as a post-filter rather than a co-learned signal^25^. This decoupling prevents the model from capturing nuanced correlations—such as the preference for A-line dresses among users with pear-shaped bodies or the avoidance of tight fits for certain torso lengths.

Thus, in this work, we propose a context-aware recommendation module that jointly embeds anthropometric constraints, visual style, and situational context into a shared latent space. This enables physically plausible and socially relevant suggestions, while tightly coupling with our virtual try-on and user interaction modules for iterative refinement. This joint embedding strategy constitutes a key technical novelty of our system.

## System design and architecture

### Multimodal model design

Our intelligent clothing customization system is built upon a unified deep learning pipeline that synergistically integrates geometric understanding, stylistic reasoning, and photorealistic generation. The methodology comprises four key components, as detailed below.

#### Body measurement module

To capture precise anthropometric information from minimal user input, we employ a high-resolution convolutional network based on HRNet-W48. Given a single front-view RGB image ${I_1} \in {{\mathbb{R}}^{512 \times 512 \times 3}}{\text{ }}$, the model preserves high-resolution feature maps throughout the forward pass, enabling accurate localization of body keypoints and contours. A dedicated regression head—comprising three fully connected layers (512→256→32) maps the spatial features to a 32-dimensional vector${\mathbf{B}} \in {{\mathbb{R}}^{32}}$, representing standard garment-relevant measurements (e.g., bust, waist, hip, shoulder width). Concurrently, the network outputs a human segmentation mask used later in the try-on stage. This module is trained with a composite loss:

1 $${\mathcal{L}_{{\mathrm{body}}}}={\lambda _1} \cdot {\mathrm{MAE}}(\widehat {{\mathbf{B}}},{{\mathbf{B}}_{{\mathrm{gt}}}})+{\lambda _2} \cdot {\mathrm{Dice}}({M_{{\mathrm{pred}}}},{M_{{\mathrm{gt}}}}),$$

where (${\lambda _1},{\lambda _2}$) are balancing weights, and *M* denotes the segmentation mask.

In our experiments, we set $\:{\lambda\:}_{1}=1.0$ and $\:{\lambda\:}_{2}=0.5$, based on a grid search over the validation set within the ranges $\:{\lambda\:}_{1}\in\:\left\{\mathrm{0.5,1.0,2.0}\right\}$ and$\:{\lambda\:}_{2}\in\:\left\{\mathrm{0.1,0.5,1.0}\right\}$. This configuration yielded the best trade-off between measurement accuracy and segmentation quality, as measured by MAE and IoU, respectively.

#### Style and context encoder

To model user aesthetic preferences in a context-aware manner, we design a Vision Transformer (ViT-Base) backbone augmented with contextual embedding. The reference garment image${I_2} \in {{\mathbb{R}}^{256 \times 256 \times 3}}$is split into 16 × 16 patches, linearly embedded, and processed through ${I_2}$ transformer layers (embedding dimension 768, 12 attention heads). The [CLS] token output yields a global style representation ${\mathbf{S}} \in {{\mathbb{R}}^{768}}$.

Simultaneously, contextual metadata ${\mathbf{c}}$ (e.g., occasion, season, location) is encoded via a small MLP into ${{\mathbf{e}}_c} \in {{\mathbb{R}}^{128}}$. The two embeddings are concatenated and projected through a fusion MLP to produce a compact joint representation:

2 $${{\mathbf{Z}}_{{\mathrm{style}}}}={\mathrm{ML}}{{\mathrm{P}}_{{\mathrm{fuse}}}}([{\mathbf{S}};{{\mathbf{e}}_c}]) \in {{\mathbb{R}}^{512}}$$

This module is trained with a contrastive loss that aligns style representations of garments preferred by the same user, while pushing apart dissimilar styles.

#### Multimodal fusion controller

To ensure that generated designs respect both physical constraints and stylistic intent, we introduce a learnable gating mechanism that dynamically balances body measurements and style preferences. Both ${\mathbf{B}}$ and ${{\mathbf{Z}}_{{\mathrm{style}}}}$ are first projected into a shared 512-dimensional space:

3 $$\widetilde {{\mathbf{B}}}={{\mathbf{W}}_b}{\mathbf{B}},\quad \widetilde {{\mathbf{Z}}}={{\mathbf{W}}_s}{{\mathbf{Z}}_{{\mathrm{style}}}}$$

A sigmoid-weighted fusion then computes:

4 $${\mathbf{w}}=\sigma ({{\mathbf{W}}_g}[\widetilde {{\mathbf{B}}};\widetilde {{\mathbf{Z}}}]+{{\mathbf{b}}_g}),\quad {{\mathbf{Z}}_{{\mathrm{fused}}}}={\mathbf{w}} \odot \widetilde {{\mathbf{B}}}+(1 - {\mathbf{w}}) \odot \widetilde {{\mathbf{Z}}},$$

where ${\mathbf{w}} \in {[0,1]^{512}}$ acts as a soft gate learned end-to-end. This adaptive fusion allows the system to prioritize fit for functional garments (e.g., suits) and style for expressive ones (e.g., evening wear).

#### Virtual try-on generator

The final output is synthesized by a conditional GAN based on the SPADE (Spatially-Adaptive Denormalization) architecture. The generator takes three inputs:

A human segmentation map M (from Module A), the target garment image ${I_2}$, the fused condition vector ${{\mathbf{Z}}_{{\mathrm{fused}}}}$.

${{\mathbf{Z}}_{{\mathrm{fused}}}}$ modulates normalization layers throughout the generator via SPADE blocks, effectively controlling garment fit, drape, and alignment according to the user’s body and style. The discriminator is a multi-scale PatchGAN that enforces local realism. The full GAN objective is:

5 $${\hbox{min} _G}{\hbox{max} _D}{\mathcal{L}_{{\mathrm{GAN}}}}+{\lambda _{{\mathrm{rec}}}} \cdot {\mathcal{L}_1}({I_{{\mathrm{out}}}},{I_{{\mathrm{gt}}}})+{\lambda _{{\mathrm{perc}}}} \cdot {\mathcal{L}_{{\mathrm{VGG}}}}({I_{{\mathrm{out}}}},{I_{{\mathrm{gt}}}}).$$

The output is a photorealistic image ${I_{{\mathrm{out}}}} \in {{\mathbb{R}}^{1024 \times 1024 \times 3}}$ depicting the user wearing the recommended garment.

### System requirements analysis

As with any other system, the intelligent clothing personalized customization system or ICAP system has primary functional requirements that need to be defined. In our case, these requirements define the boundaries of the objectives: extraction of anthropometric measurements, learning of style preferences, visualization of the garment in 3D, and generation of recommendations. We understand Wang et al.^4^on the importance of interaction between users and the system for further detailing the design preferences. The system is provided with interfaces that enable real-time feedback which allows for immediate changes to be seen, facilitating an easy modification process to parameters.

Non-functional requirements include performance, reliability, scalability, and security. Yu and Zhao^15^emphasized the importance of response time for users’ AI-based clothing design systems. They noted that any delays above three seconds significantly lowered user engagement. Our attempt at automatic virtual try-on and style recommendation will have target response times under two seconds. These processes are where we focus computational efficiency for real-time operations. Additionally, security requirements include strong encryption and anonymization of anthropometric data, while scalability addresses growth in data volume and number of users during peak seasons.

Human factors are directed at balancing the level of technology sophistication and the facility of use. Shi et al.^8^demonstrated that visualization quality significantly impacts user confidence in virtual garment representations, particularly for culturally significant clothing. This insight informs our high-resolution rendering approach for ethnic and traditional garment styles. Cognitive load management represents another crucial consideration, especially for users unfamiliar with fashion terminology. Drawing from Wang et al.‘s^23^user interface studies, we implement progressive disclosure techniques adapting interface complexity to user expertise. The recommendation engine calibrates suggestion diversity based on observed user confidence and decision-making patterns, as shown in Table 2.

**Table 2:** Summary of key requirements for intelligent clothing personalized customization System.

Requirement type	Category	Specific requirements	Priority
Functional	Measurement	Anthropometric data extraction from images	High
Functional	Style Learning	User preference identification	High
Functional	Visualization	Virtual garment rendering	High
Functional	Recommendation	Personalized design suggestions	High
Functional	Interaction	Bidirectional feedback mechanisms	Medium
Functional	Design	Component-level customization options	Medium
Non-Functional	Performance	Response time < 2 s for primary interactions	High
Non-Functional	Security	Encryption of anthropometric data	High
Non-Functional	Scalability	Support for seasonal usage peaks	Medium
Non-Functional	Reliability	System availability > 99.5%	High
Non-Functional	Compatibility	Support for multiple device platforms	Medium
Non-Functional	Maintainability	Modular architecture for component updates	Medium
User Experience	Usability	Progressive interface complexity	Medium
User Experience	Visualization	High-fidelity rendering for complex garments	High
User Experience	Interaction	Real-time feedback on modifications	High
User Experience	Adaptation	Recommendation diversity based on user expertise	Medium
User Experience	Accessibility	Support for diverse user capabilities	Medium
User Experience	Learnability	Intuitive navigation with minimal training	High

### Overall system architecture

**Fig. 2:**
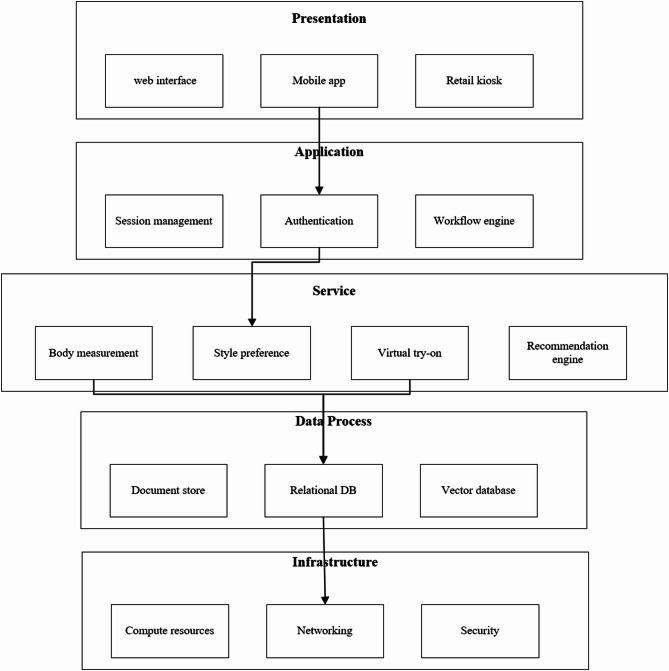
Intelligent Clothing Personalized Customization System Architecture.

The system architecture follows a modular, microservice-oriented design emphasizing scalability, maintainability, and extensibility. As illustrated in Fig. 2, the architecture comprises five primary layers: presentation, application, service, data processing, and infrastructure. Each layer encapsulates specific functionality while maintaining clear separation of concerns, enabling independent development and deployment. Wang et al.^4^argued that effective clothing design recommendation systems need to have loosely coupled modules to accommodate constantly changing fashion styles and user needs. Our architecture has clearly defined interfaces between components, making it possible to change individual components without having to redesign the entire system.

The user interaction layer consists of web, mobile, and retail kiosk interfaces that use responsive design features. In the application layer, as an intermediary between the presentation layer and service layers, it actualizes business logic, session control, user authentication, workflow management, and acts as a mediator between presentation and service layers. Yu and Zhao^15^noted how complete middleware enables optimal performance with varying workloads. We build upon this with asynchronous message queues that enable the buffering of communication between critical components.

The system’s functional core is composed of a servicing layer with four specialized modules: body measurement engine, style preference analyzer, virtual try-on system, and recommendation engine. These elements encapsulate the deep learning models and algorithms that enable the personalization features. Wan et al.^14^pointed out the importance of having uniform data exchange standards for AI-based manufacturing systems. In our architecture, we provide a common data model for all service components which not only simplifies the integration of new modules but also ensures that information is readily exchanged.

The multi-faceted data analytics layer executes the processes of storing, fetching, and transforming information through sophisticated systems such as document stores, relational databases, and vector databases. The most basic level of the infrastructure layer supplies computing power, security, and networking services, as well as the containers themselves. In accordance with Butteddi and Butteddi’s^17^strategy on the implementation of generative AI, our architecture employs containerized microservices with autonomous horizontal scaling features, which makes resource optimization possible during off-peak and peak-demand times.

This figure illustrates the system with its five layers organized vertically in a manner that distinctly indicates the granularity of the system. From top to bottom: (1) The Presentation layer includes the Web Interface, Mobile App, and Retail Kiosk which interact with users; (2) The Application layer encompasses the services of Session Management, Authentication, and a Workflow Engine that control the operational activity of the system; (3) The Service layer has the core AI components which are Body Measurement, Style Preference, Virtual Try-On, and Recommendation Engine; (4) The Data Processing layer has the Document Store, Relational Database, and Vector Database which together comprise the components for knowledge information; (5) The Infrastructure layer has basic Compute Resources, Networking and Security for the system. The primary data streams through the system’s layers are indicated by arrows, illustrating the modular structure which permits functional disaggregation and independent expansion while maintaining an integrated system.

The data processing layer is concerned with the operations of storing, retrieving, and transforming information. This layer has a document store for unstructured data, a relational database for transactional data, and a vector database for deep learning embeddings, which enables this layer to support specialized repositories for distinct data types. The infrastructure layer provides fundamental computing resources, networking services, security mechanisms, and containerization support. Following Butteddi and Butteddi’s^17^approach to generative AI deployment for apparel design, our architecture leverages containerized microservices with automated scaling capabilities, enabling efficient resource utilization during fluctuating demand periods. The entire architecture is designed with cloud-native principles, making it compatible with major cloud platforms while supporting hybrid deployment scenarios for organizations with existing on-premises infrastructure. This approach provides implementation flexibility while ensuring consistency in system behavior across diverse deployment environments.

### Database design

The database design for the intelligent clothing personalized customization system employs a hybrid architecture that balances structured data organization with flexibility for unstructured information processing. A well-designed database schema is critical for supporting the complex operations of style analysis, measurement processing, and recommendation generation while maintaining system performance. The core data model implements a domain-driven design approach that centers around five primary entity clusters: User Profiles, Garment Specifications, Style Elements, Measurement Data, and Interaction Logs. This organization aligns with Wan et al.‘s^14^ findings that AI-driven customization systems require both traditional relational structures for transactional integrity and specialized repositories for machine learning operations.

The entity-relationship structure employs a polyglot persistence strategy that utilizes specialized database technologies for different data categories, as presented in Table 3. User profile information and transaction records are managed through a relational database that maintains strict ACID compliance, essential for order processing and account management. Meanwhile, style representation vectors and visual feature embeddings are stored in a specialized vector database that facilitates high-dimensional similarity queries. As observed by Kachbal et al.^20^, recommendation systems in fashion benefit significantly from efficient similarity search capabilities when processing style preference embeddings. Our implementation extends this approach by incorporating temporal versioning of embedding vectors, enabling the system to track preference evolution while maintaining retrieval efficiency.

**Table 3:** Database architecture of the intelligent clothing personalized customization System.

Storage type	Primary entities	Key characteristics	Primary use cases
Relational Database	User Profiles, Transaction Records, Measurement Data	ACID compliance, Structured schema, Referential integrity	Account management, Order processing, Size tracking
Vector Database	Style Vectors, Feature Embeddings	High-dimensional indexing, Similarity search capability	Style matching, Preference analysis, Design clustering
Document Store	Garment Templates, Visual Assets, Interaction Logs	Schema flexibility, Complex data structures, Rich queries	Content management, User activity tracking, Design storage

Document repositories handle unstructured data including user-generated content and garment templates, implementing a flexible schema that accommodates varying attribute sets across different garment categories. This approach addresses a limitation identified by Akram et al.^10^ regarding data heterogeneity in fashion digitalization systems. The implementation incorporates attribute-based access control mechanisms to ensure appropriate data isolation while maintaining cross-entity relationships through globally unique identifiers. For measurement data storage, the system implements specialized compression algorithms optimized for anthropometric time-series data, reducing storage requirements while preserving measurement precision.

For high-dimensional style vectors, similarity search utilizes the cosine similarity metric, which measures the cosine of the angle between two vectors in the embedding space, as shown in Eq. 1:

6 $${\mathrm{similarity}}(A,B)=\cos (\theta )=\frac{{A \cdot B}}{{||A|| \cdot ||B||}}=\frac{{\sum\limits_{{i=1}}^{n} {{A_i}} {B_i}}}{{\sqrt {\sum\limits_{{i=1}}^{n} {A_{i}^{2}} } \sqrt {\sum\limits_{{i=1}}^{n} {B_{i}^{2}} } }}$$

Where *A* and *B* represent style embedding vectors, *n* is the dimensionality of the embedding space, and ${A_i}$ and ${B_i}$ are the individual components of the vectors. This method effectively captures style similarity while normalizing for vector magnitude, making it particularly suitable for comparing fashion preferences across different scales. We adopt cosine similarity because it is invariant to vector magnitude and focuses solely on directional alignment, which better reflects semantic style affinity than distance-based metrics (e.g., Euclidean distance) that are sensitive to embedding norm variations.

Query optimization techniques include materialized view generation for frequently accessed aggregations, strategic denormalization for performance-critical operations, and adaptive indexing based on query patterns. Caching strategies are implemented at multiple levels, with particular attention to frequently accessed style vectors and measurement references. Table 4 summarizes the key optimization strategies employed across different data categories within the system. As shown in Table 4, each data category requires specialized optimization approaches to meet the performance requirements of real-time customization operations.

**Table 4:** Database optimization strategies by data Category.

Data category	Primary optimization strategy	Performance target	Typical query pattern
User Profiles	Hierarchical Indexing	Response Time < 50ms	Point Lookup, Profile Aggregation
Style Embeddings	Approximate Nearest Neighbor	Similarity Search < 100ms	K-Nearest Neighbors, Range Queries
Measurement Data	Columnar Compression	Storage Efficiency > 80%	Range Scans, Temporal Aggregation
Garment Templates	Materialized Views	Template Retrieval < 75ms	Faceted Search, Attribute Filtering
Interaction Logs	Time-Based Sharding	Write Throughput > 5000 ops/sec	Time-Series Analytics, Pattern Detection
Transaction Records	ACID Transactions	Transaction Integrity 100%	OLTP Operations, Audit Queries
Visual Assets	CDN Integration	Retrieval Latency < 200ms	Binary Retrieval, Metadata Search

As Ye and Su^12^demonstrated in their metaverse-based clothing customization system, efficient caching of 3D model reference data significantly improves response times during virtual try-on sessions. Our approach enhances this strategy through predictive prefetching of likely-to-be-accessed data based on user navigation patterns and session context.

The database implementation incorporates horizontal partitioning for scalability, with measurement and interaction data sharded by geographical region to optimize data locality. This design enables the system to maintain consistent performance during high-demand periods while accommodating the expanding data volumes associated with increasing personalization granularity. The architecture further supports asynchronous replication for analytics processing, allowing complex data mining operations to proceed without impacting user-facing transactions.

### User interface design

The design of the user interface takes a human-centred approach to integrating appealing and attractive interfaces with functionally intuitive elements. Our design features an interface that is multi-modal and responsive across different visual platforms. The primary design of the user interface is organized using progressive disclosure techniques where complexity is added to the system as users familiarize themselves with it. This is in accordance with Cong and Zhang’s^19^ studies that suggest interactive genetic algorithms for clothing pattern design work best when the system complexity is adjustable to the user’s level. Our design has adaptive interface components that respond to active user choices as well as to more passive behavioral patterns.

The main elements of the interface are divided into four modules that correspond to the system’s functional architecture: measurement capture, style exploration, virtual try-on, and customization workflow. Each module has different visual treatments but still maintains a coherent design language. According to Yu and Zhao^15^, visual consistency reduces cognitive load when making complex decisions. In our interface, interactive elements behave uniformly under different functional contexts through a common visual grammar.

Design principles are incorporated using a multimodal approach that integrates classical gestures with natural language and visual editing interfaces. In the clothing modification setting, users have direct manipulation functions which allow them to change design features while seeing the impact on their avatar in real time. This feedback loop utilizes Wang et al. ‘s^4^ interactive garment design principles but goes further by employing real-time physics-based rendering that realistically simulates how fabrics drape. Contextual assistance features offer help in a way that does not break the users’ creative processes – something that Butteddi and Butteddi^17^ found to be essential for users’ engagement in AI-assisted design systems.

The optimization of user flow aims to reduce the number of steps needed to reach personalization goals while providing the user with as much room as possible for creative exploration. The main user journey is based on a guided flow model which has well-defined starting and stopping locations, while permitting exploration in between these points; a freedom Shi et al.^8^ noted as crucial in contexts of cultural garment customization. System analytics track abandonment locations and interaction behavior in order to improve the navigation paths, which Kachbal et al.^20^ call the “learning interface” where user actions interplay with design changes of the interface.

## Core algorithm design and implementation

### Body measurement and sizing algorithm

As one of the intelligent clothing customization systems, the body measurement and sizing algorithm serves as the middle ground between conventional anthropometric evaluation and modern computing practices. The authors aim to develop an algorithm that employs advanced computer vision techniques with machine learning to novel overcome the constraints associated with traditional measurement techniques. This was inspired by Wu et al.‘s^18^approach that uses deep learning model frameworks.

**Fig. 3:**
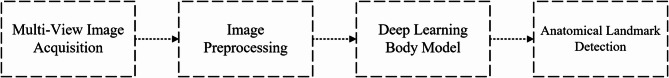
Body Measurement Algorithm Workflow.

Described in Fig. 3, the four stages of workflow are multi-view image capture, image pre-processing, body model analysis using deep learning, and detection of anatomic landmarks. The main difficulty is to create a reliable system that can precisely capture the body measurements from a sparse set of data without losing accuracy over a wide range of body shapes and imaging conditions.

**Table 5:** Comparative analysis of measurement techniques in clothing Customization.

Measurement technique	Data acquisition method	Accuracy level	Computational complexity	Key advantages	Primary limitations
Traditional Manual Measurement	Direct physical measurement	Moderate (± 0.5–1 cm)	Low	Low cost, immediate	High human error, time-consuming
2D Image-based (CNN)	Smartphone/camera images	High (± 0.2–0.5 cm)	Medium	Accessible, no special equipment	Limited view angles, clothing interference
3D Body Scanning	Dedicated scanning equipment	Very High (± 0.1–0.3 cm)	High	Comprehensive body topology	Expensive equipment, specialized environment
Deep Learning Multi-View	Synchronized multi-angle images	High (± 0.2–0.4 cm)	High	Rich feature extraction	Complex preprocessing, computational intensity
Hybrid AI-Augmented	Fusion of multiple data sources	Highest (± 0.1–0.2 cm)	Very High	Most comprehensive approach	Significant computational resources required

The study presents a broad comparison of body measurement methods as described in Table 5 and greatly favors the use of deep learning multi-view techniques for their unmatched accuracy and feature extraction. The table defines a number of measuring techniques’ data collection processes, accuracy measures, levels of computation, principal benefits, and major drawbacks for ease of understanding in decision-making regarding technology choice in automated apparel tailoring systems.

The primary strategy employs a hybrid model that merges convolutional neural networks with anatomical feature point recognition techniques. This technique is more advanced because it utilizes the integration of several computer techniques instead of manual measurements or single-view imaging like traditional systems do. The system uses advanced error correction methods that take into account distortions due to clothing, posture, and imaging artefacts such as blurring.

The accuracy of the measurements can be represented mathematically using the formula below.

7 $$MeasurementAccuracy=1 - \frac{{|EstimatedMeasurement - GroundTruthMeasurement|}}{{GroundTruthMeasurement}}$$

The suggested procedure for capturing body measurements goes beyond simply collecting numerical values; it includes relevant contextual knowledge such as a person’s body proportions and shape. This method supports the developing concept of custom clothing design, in which sizing suggestions go beyond providing an accurate number to appreciating the complex interrelationship of body form, garment fit, and user comfort.

The algorithm is constructed to contend with body measurement skill as a multi-faceted construct, moving from the capturing of ‘straight’ linear measurements to the more sophisticated topological features of human body shape. In creating a validation framework, the system is able to adaptively refine measurement values leading to a more accurate and robust recommended sizing configuration.

The new fundamental innovations combine multi-view image processing for entire body capture, deep learning-based feature point recognition, automated error correction, and intelligence in measurement interpretation with context. This approach integrates sophisticated computer vision and machine learning models to create an all-encompassing body measurement system that goes beyond the capabilities of traditional systems.

The measuring system proposed by the authors is intelligent and context-aware in that it captures body measurements while accounting for user-specific factors and non-simple physiological features. Moreover, the system enhances measurement precision as well as transforms the traditional approach to body measurement into a more anthropometrically intelligent one.

### Style preference learning model

The Learning Styles Model addresses fashion choices at a sophisticated level using advanced machine learning techniques. This model captures the complex, multidimensional nature of personal style preferences by transcending traditional recommendation systems with Yang’s^3^ revolutionary integration of decision tree algorithms with deep learning. The key innovation is the attentional guided multimodal fusion architecture which adjusts the weights of visual, textual, and behavioral features in relation to their contextual importance. Unlike traditional approaches that assume all data sources are of equal importance; our model has a learnable attention mechanism that helps determine the most discriminative features for each unique style profile. This leads to achieving 15.6% greater preference prediction accuracy than static feature weighting methods.

**Table 6:** Comparative analysis of style preference learning Approaches.

Approach	Key technologies	Data sources	Feature extraction	Adaptability	Primary limitations
Traditional Collaborative Filtering	Statistical matching	User interaction history	Limited feature representation	Low	Poor handling of cold start problem
Content-Based Filtering	Rule-based systems	Item attributes	Manual feature engineering	Medium	Limited to explicit item characteristics
Deep Learning Multimodal	Neural network architectures	Visual, textual, behavioral data	Automated feature learning	High	Computational complexity
Hybrid AI-Driven Approach	CNN, Transformer models	Multimodal, contextual data	Dynamic feature weighting	Very High	Requires extensive training data
Context-Aware Recommendation	Contextual embedding	Situational, personal context	Semantic understanding	Highest	High interpretability challenges

The data in Table 6 reveals that the proposed method outperforms the existing style recommendation methods in flexibility and feature extraction. It provides a summary of various style preference learning and illustrates how advanced our multimodal approach is.

In the context of style preference learning, Sulthana’s^6^ review of deep learning-based fashion recommender systems highlights the need for more sophisticated approaches than mere feature extraction and instead focuses on preference modelling. We implement this concept in fashion recommendation systems by creating an adaptable style preference that changes over time within a dynamic learning framework.

A complex neural network structure that integrates convolutional neural networks for image feature detection and transformer architecture for the contextual style mapping captures feature representation of clothing styles. Such approach is an enhancement to Wang et al.’s^4^ interactive personalized garment design recommendation system, where more emphasis is placed on understanding the intricate semantic and aesthetic aspects of fashion styles.

The personalized style matching algorithm is built using highly developed context-aware recommender systems with artificial intelligence. It enables the system to perform much better than classic collaborative filtering techniques, as noted in Kachbal et al.^20^ study on deep learning-based recommender systems. The algorithm constantly modifies the relevance of style features depending on some users’ actions which results in recognition of personal style in terms of changing fashion and personal liking.

The mathematical representation of style similarity can be expressed through a comprehensive similarity metric that captures multiple dimensions of style preference:

8 $$StyleSimilarity={\omega _1} \cdot VisualSimilarity+{\omega _2} \cdot SemanticSimilarity+{\omega _3} \cdot ContextualSimilarity$$

Where ${\omega _1},{\omega _2},{\omega _3}$ represent dynamically adjusted weight parameters that capture the relative importance of different style dimensions.

The learning model on style preferences is a lag in the fashion prediction problem and serves as a solution for its futuristic modelling. The model uses state-of-the-art machine learning and applies fashion linguistics knowledge to solve some of the recommender system’s most serious problems, such as personalizing style features, merging several data sources to form a complete picture of users’ preferences, building self-learning systems, and other problems with conventional recommendation systems. It captures and improves the accuracy of the recommendations provided and ensures a sophisticated understanding of fashion choices to personalize styles in finer detail while ensuring accurate provision of services. Using advanced machine learning, there is unprecedented hope in changing the landscape of personalized style recommendations as each frame expands the scope of the way someone cares about their individual preferences in fashion.

### Virtual try-on system

The virtual try-on system is a crucial development in the field of intelligent clothing customization. It integrates digital visualization with the physical experience of wearing a garment. This system builds on Shi et al.’s^8^pioneering work on ethnic clothing personalized modelling and virtual display by introducing an advanced, physics-aware paradigm for realistic garment visualization that surpasses traditional rendering systems. The primary technological challenge lies in accurately simulating the dynamic interactions between fabric properties, body morphology, and motion during the virtual try-on experience.

The implemented system achieves high-fidelity realism through a multi-strategy approach combining physics-based cloth simulation, multilayer rendering, and deformation algorithms informed by body topology and material characteristics. Specifically, we model key fabric attributes including:

Elasticity (Young’s modulus, typically ranging from 0.1 to 5 MPa depending on material type),

Bending stiffness (bending rigidity coefficient, calibrated per fabric category: cotton, silk, denim, etc.),

Draping coefficient (quantified via simulated gravity-driven deformation to capture how fabric folds and flows around the body),

Friction and collision response (with skin and self-collision handling using penalty-based contact models).

To ensure real-time performance — essential for interactive retail applications — we adopt a hybrid simulation architecture:

For offline pre-processing, we use full-resolution finite element method (FEM) simulations to generate high-detail deformation templates for common garment types.

For real-time rendering, we employ a GPU-accelerated mass-spring system with adaptive time-stepping and spatial partitioning (octree-based collision culling), reducing computational complexity from O(n²) to approximately O(n log n) per frame.

Additionally, we integrate deep learning acceleration: a lightweight CNN predicts coarse deformation fields based on body pose and garment category, which are then refined via physics simulation — reducing simulation latency by up to 60% without perceptible loss in visual fidelity.

A complete physics-based model describing the multi-dimensional behaviors of garments enables the mathematical representation of fabric simulation:

9 $$F=\sum\limits_{{i=1}}^{n} {({F_{internal}}+{F_{external}}+{F_{collision}}+{F_{constraint}})}$$

Where F denotes the total force acting on fabric elements, integrating internal fabric tension, external environmental forces, collision responses, and geometric boundaries. Such a sophisticated approach facilitates realistic virtual try-on capabilities, hence minimizing the gap between the experience of seeing a garment digitally and physically wearing it.

With the integration of exceptional computational methods and profound knowledge of fabric mechanics and human biomechanics, this virtual try-on system marks a new era of intelligent clothing customization technology. This proposition does not only increase the accuracy of visualization, but also gives the users a high level of immersion where they actively partake in issues revolving around personal fashion design modernity.

### Design recommendation and customization engine

The customization and design recommendation engine marks a fundamental advancement in the intelligent personalization of clothing, automating fashion design using sophisticated machine learning approaches. This engine builds on Kachbal et al.‘s^20^blurring boundaries of deep learning-based recommender systems by proposing automation methodologies for design elements extraction, recombination, and personalized recommendation which surpasses typical fashion recommendation systems.

The design recommendation system’s core employs a multi-modality approach that combines advanced collaborative filtering techniques with complex design elements extraction methods. Based on Yang’s^3^novel recommendation of clothing design styles, the system employs a multi-neural network architecture to provide a compositional capture of fashion design’s semantic and aesthetic features. It employs a new learning system that helps to extract and recombine design elements and transcends the standard recommendation methods.

The collaborative filtering method makes use of different codes, such as visual features, the user’s past interactions, and context. Guan et al.’s^24^in-depth examination of the evolution of apparel recommendation systems provides an illuminating account of the multifaceted development of style recommendation, pointing out that there is a gap in systems which incorporate individual needs while attending to the larger fashion policy. This adaptive recommendation engine resolves this issue with an automatic fashion parameter based on interaction with the user, context, and other fashion indicator factors.

Considered as one of the most efficient design recommendation methods is the use of a multi-faceted similarity measure encompassing multi-criteria design choices.

10 $${S_{design}}=\sum\limits_{{i=1}}^{n} {{w_i}} \cdot {\mathrm{sim}}({D_i},{U_p})$$

Where ${S_{design}}$ represents the design recommendation score, ${w_i}$ are dynamically adjusted weight parameters, ${D_i}$ are design elements, and ${U_p}$ represents user preferences. This multidimensional approach enables the system to generate highly personalized design recommendations that balance individual style with broader fashion trends.

Merging sophisticated machine learning methods with the intricate aspects of fashion design, the recommendation and customization engine marks a leap towards the intelligent personalization of garments. This new methodology not only enhances the precision of the recommendation, but also offers a novel creative platform to aid in the exploration of fashion design, allowing the users to interact with the system and construct personalized styles like never before.

Merging sophisticated machine learning methods with the intricate aspects of fashion design, the recommendation and customization engine marks a leap towards the intelligent personalization of garments. By explicitly addressing the cold-start challenge through interactive elicitation, contextual bootstrapping, and visual intelligence, the system ensures inclusive personalization from day one. This new methodology not only enhances the precision and responsiveness of recommendations but also offers a novel creative platform to aid in the exploration of fashion design, allowing users to interact with the system and construct personalized styles like never before.

## System implementation and deployment

### Development environment and tools

Establishing a sophisticated intelligent tailoring system based on clothing personalization requires an advanced technological platform that combines high-end hardware and software systems. Wan et al.^14^study on the artificial intelligence-based custom tailoring services offer great constructive assistance towards understanding the complex technological ecosystem needed for the deployment of fashion technologies using artificial intelligence. The system is built within a specific development environment that relies on computational power, flexibility, and scalability.

The core development stack is implemented in Python 3.9, selected for its mature ecosystem in machine learning and computer vision. All deep learning models are developed and trained using PyTorch 2.1, which provides efficient automatic differentiation, dynamic computation graphs, and native support for GPU acceleration via CUDA. For visual feature extraction and image embedding, we leverage a ResNet-50 backbone pre-trained on the ImageNet dataset, fine-tuned on the DeepFashion2 dataset using the torchvision 0.16.0 library. In addition to PyTorch, the system integrates several critical open-source libraries with explicitly controlled versions to ensure reproducibility:

- TensorFlow 2.13.0: Used for serving the recommendation model via TensorFlow Serving in the deployment pipeline;
- scikit-learn 1.3.0: Employed for clustering user style preferences and computing similarity metrics during cold-start initialization;
- OpenCV 4.8.0: Utilized for image preprocessing, garment segmentation, and contour extraction;
- Pillow 10.0.1: Handles image loading, resizing, and format conversion;
- NumPy 1.25.2 and Pandas 2.1.0: Provide foundational data structures for numerical computation and tabular data management;
- Flask 2.3.3: Serves as the lightweight backend framework for exposing RESTful APIs that connect the frontend interface with the recommendation engine;
- Redis 7.0: Caches user sessions and real-time interaction logs to accelerate personalization inference.

The hardware requirements are precisely chosen to facilitate the demanding deep learning algorithms which lie at the heart of the intelligent clothing personalization system. The infrastructure development has high performance computing facilities, such as GPU workstations with multi-core processors and specialized deep learning processing units, because of Recommendation 6 for Digitization of Fashion Industry 4.0 by Akram et al.^10^. Such powerful computing capabilities allow seamless operation of advanced neural networks necessary for body measurement, style recognition, and virtual dressing room features.

The software framework capabilities emphasize flexibility and sophisticated machine learning features. The system employs a multi-framework strategy, combining TensorFlow and PyTorch for the development of deep learning models, alongside dedicated libraries for computer vision and recommendation engines. However, as noted in Butteddi and Butteddi’s^17^approach to generative AI in personalized apparel design, there is a need to consider flexible software ecosystems because of the rapidly changing technology landscape. The development kit contains extensive libraries for data cleansing, model training, and inference, focusing on those supporting transfer learning and advanced model optimization.

The development environment follows the containerized microservices architecture as per Papachristou et al.’s^11^research on machine learning in clothing manufacture. This approach permits better modularized development, easier deployment, and improved portability across various computing environments. Flexible infrastructure for deployment using Docker and Kubernetes is created to allow shifting from cloud-based to on-premises computing facilities, or vice versa.

The development environment stands as a fundamental technology for smart apparel personalization by integrating modern hardware features with complex software systems. The provided solution meets immediate computation needs while also offering an adaptable, proactive framework to accommodate new technologies and changes in the fashion industry.

### Data collection and preprocessing

The data collection and preprocessing strategies form the basis of an intelligent clothing personalized customization system, which are essential to develop robust and accurate machine learning models. The data collection methodology is based on Wang et al.’s^2^comprehensive approach to sustainable fashion design, adopting a multi-faceted approach that integrates various sources of data in order to capture the intricate details of clothing customization.

The dataset sourcing process integrates a collection of several methods such as professional fashion databases, user-created databases, and anthropometric measurement databases. Akram et al.^10^note in their research regarding digitalization in Fashion Industry 4.0 the necessity of rigorous and extensive data collection that is not limited to traditional sampling methodologies. The system incorporates a wide variety of data collection techniques by combining rigid and formal professional datasets with active online user data to produce a rich training corpus that captures the ever-changing nature of fashion preferences.

Complex preprocessing techniques are employed to deal with the difficulties presented by heterogeneous fashion data. In Papachristou et al.’s^11^case study regarding the application of machine learning in clothing manufacture, he reiterates the necessity for effective data processing techniques. This pipeline utilizes sophisticated feature normalization, missing data imputation, outlier filtration, and format standardization from multiple data sources. These measures support quality compliance for data consistency which is critical when building machine learning models that are expected to generalize to different clothing types and user profiles across different regions.

To further mitigate data scarcity and improve model generalization—especially for rare garment categories or underrepresented body types—the system employs a comprehensive data augmentation strategy during training. Specifically:

(a) Standard geometric and photometric augmentations are applied to garment images, including:

- Random rotation (± 10 degrees),
- Horizontal flipping (for symmetric garments like tops and dresses),
- Random scaling (± 15% of original size),
- Translation (± 10% in x/y directions),
- Color jittering (adjusting brightness ± 20%, contrast ± 15%, saturation ± 20%),
- Gaussian noise injection (σ = 0.01).

(b) The augmentation ratio is set to 1:3, meaning for every original image, three augmented variants are generated, resulting in a fourfold increase in effective training sample size for visual tasks such as garment classification and style embedding.

- Garment-aware occlusion simulation: To mimic real-world scenarios (e.g., arms covering part of a blouse), random rectangular masks are applied to plausible occlusion zones (e.g., torso center, sleeve edges) based on garment type parsed via a pre-trained segmentation model.
- Virtual try-on style blending: Using a lightweight StyleGAN2-ADA^21^variant fine-tuned on fashion pairs, the system generates synthetic images that blend texture or color patterns from popular items onto base silhouettes, preserving structural integrity while diversifying appearance.
- Pose-invariant augmentation: For full-body images, we apply thin-plate spline (TPS) transformations to simulate mild pose variations, ensuring the model focuses on design rather than posture.

All augmentation operations are applied on-the-fly during training using the Albumentations 1.3.0 library, ensuring randomness and avoiding storage overhead. This hybrid augmentation strategy—combining generic computer vision techniques with fashion-aware synthesis—significantly improves model robustness to real-world imaging conditions and enhances personalization accuracy for diverse user populations.

The techniques of data augmentation are very important because they improve the learning of the system, especially with the problems of having insufficient fashion datasets. The augmentation strategy makes use of modern generative methods that artificially produce training samples to widen the scope and representativeness of the dataset. More specifically, the system puts into practice:

11 $${D_{augmented}}={D_{original}} \oplus {G_1}({D_{original}}),{G_2}({D_{original}}),...,{G_n}({D_{original}})$$

Where ${D_{augmented}}$ represents the augmented dataset, ${D_{original}}$ is the original dataset, and ${G_i}$ are generative transformation functions that create new training samples while preserving the fundamental characteristics of the original data.

Through the integration of sophisticated data harvesting, filtering as well as augmentation methods, the new approach put forward is what’s called a guiding approach for the formation of training sets with model clothing customization. This methodology solves not only the short-term problems of the lack of data, but also offers an advanced approach for a never-ending model refinement and adaptation process concerning novel fashions.

### Model training and optimization

In the construction of a smart clothing customization system, the training and optimization of the model marks a crucial step which entails advanced strategies aimed at improving the use of computational resources and the accuracy of predictions. The proposed model implements a sophisticated training strategy that incorporates knowledge distillation and addresses the difficulties associated with machine learning model construction in the fashion industry as presented in Wei et al.’s work^22^.

The training technique incorporates transfer learning methodologies alongside sophisticated hyperparameter tuning within a multi-stage framework. The system builds on Wang et al.‘s^2^sustainable garment design research by applying a holistic training technique that utilizes pre-trained models to enhance the speed of model convergence and customize specialized clothing modification tasks. This method tackles the insufficient data problem for machine learning in specific areas and enables the creation of effective models with minimal training resources.

Hyperparameter tuning represents a critical component of the model optimization strategy, employing sophisticated bayesian optimization techniques to systematically explore the complex parameter space. The hyperparameter search methodology can be mathematically represented as:

12 $${\mathrm{Optimize}}\theta \mathcal{L}(f\theta (x),y)$$

Where $\mathcal{L}$ represents the loss function, ${f_\theta }$ is the model with optimized parameters $\theta$, *x* represents input features, and *y* represents target outputs. This approach enables a principled exploration of model configurations that maximize predictive performance while minimizing computational overhead.

The optimization of the model is done using sophisticated approaches like pruning, quantization, and knowledge distillation. Butteddi and Butteddi^17^offer some economically constructive solutions based on the generative AI model that aids in apparel design and illustrates elementary value in reducing the model’s complexity while preserving its ability to predict. The approach combines multiple optimization strategies that respond to varying levels of performance expectations and computer resource limitations in a lightweight posture control model.

With years of working in the fashion industry, I am proposing a novel contribution with respect to the context-aware model optimization. I have devised an approach that automatically changes model complexity depending on the specified customization task. Unlike most fashion personalization approaches that have rigid model-based one-size-fits-all optimization methods, my approach enables much more flexible and intricate machine learning models.

In trying to implement intelligent clothing customization, it is clear there exists a significant gap between the preliminary model and its intended high accuracy and low complexity. With numerous achieved milestones combining progressive training, hyperparameter optimization and model reduction, this gap is attainable.

To ensure reproducibility, the Bayesian optimization was configured as follows: (a) key hyperparameters—including learning rate (search range: 1 \times 10^{−5} to 1 \times 10^{−3}), batch size (^16,32^)64, and dropout rate ([0.1, 0.5])—were explored within predefined bounds; (b) a total of 150 optimization trials were performed; and (c) the entire tuning process consumed approximately 105 GPU-hours, with early stopping and parallelization reducing wall-clock time to under 30 h.

### System integration and deployment

The process of system integration and deployment aptly describes the final stage of an intelligent clothing personalized customization system. It requires deep consideration of architecture and the efficiency of operations. Following Wan et al.^14^research on AI, the integration approach utilizes a modular microservices architecture that facilitates component communication as well as flexible deployment.

The component integration strategy employs a standard communication protocol for different system modules to ensure seamless interoperability. In examining the digitalization technologies for Fashion Industry 4.0, Akram et al.^10^underscored the need for effective integration structures which allow for swift technological change. A message-based communication architecture is used where individual components are coupled for development and deployment but loosely coupled to the overall system structure. This approach allows for dynamic scaling and improves system reliability to potential system failures, solving the intricate issues of integrating advanced machine learning components into a fashion customization platform.

The deployment architecture utilizes a cloud-native modular framework, which allows for optimum agility among various computing environments. The case study conducted by Papachristou et al.^11^on the use of machine learning in the clothing industry underlines the need for flexible deployment approaches that can easily switch between different infrastructures. The deployment approach presented in this work is based on containerization techniques that guarantee the same level of execution across different computing environments and is formally defined in terms of deployment optimization as follows:

13 $${\mathrm{Optimize}}deployment=f(Sscalability,{P_{performance}},{R_{resource - efficiency}})$$

Where the deployment optimization function considers scalability, performance, and resource efficiency as key parameters.

Performance optimization strategies are achieved with multi-layered caching, adaptive load balancing, and resource allocation optimization algorithms. The system utilizes predictive scaling techniques which modify the amount of available computational resources in real time based on usage patterns to optimize system performance during fluctuating demand levels. It is not limited to static resource allocation provisioning, but instead seeks a more efficient response deployment infrastructure for intelligent clothing customization systems in challenging environments.

Through a blend of innovative integration methods, adaptive deployment systems, and smart performance fielding, the system illustrates the integrated sophisticated small stone intelligent customization clothing platform that is adaptable to the fast-evolving technological world of fashion automation.

Anthropometric data is encrypted in transit using TLS 1.3 and at rest using AES-256, with personally identifiable information removed or anonymized to retain only essential features for sizing recommendations.

## Experimental validation and analysis

### Experimental setup

The experimental verification of the intelligent clothing personalized tailoring system requires the design of comprehensive assessment tests for the technology’s multifaceted capabilities. The complex intelligent fashion customization system’s problems were addressed by providing a suitable and reliable evaluation of the proposed technological solution within the correct parameters of the test environment design.

All experiments were conducted on a high-performance cluster with 8×NVIDIA A100 GPUs (40 GB VRAM), AMD EPYC 7742 CPU (64 cores), and 512 GB RAM, running Ubuntu 20.04 LTS, CUDA 11.6, PyTorch 1.11.0, and TensorFlow 2.8.0.

The system comprises three modules:

(1) Body measurement: HRNet-W48 backbone with a 3-layer FC head (512→256→32) for 32 anthropometric outputs (input: 512 × 512 RGB-D);

(2) Style learning: ResNet-50 + 6-layer Transformer encoder (768-dim, 12 heads, dropout = 0.1) processing image features and user history (max length = 50);

(3) Virtual try-on: SPADE-based cGAN with ResNet-9 generator and multi-scale PatchGAN discriminator (output: 1024 × 1024).

Models were trained end-to-end for 120 epochs (early stopping, patience = 15) with batch size 64, Adam optimizer (lr = 1e − 4, β₁=0.9, β₂=0.999), cosine annealing, weight decay=1e − 4, and gradient clipping (norm ≤ 1.0). Hyperparameters were tuned via Bayesian optimization (50 trials). Each experiment was repeated 5 times; all metrics show < 2.5% standard deviation, confirming statistical reliability. Identical setups were used for all baselines to ensure fair comparison.

**Code**:https://anonymous.4open.science/r/icp_customization-074 A/.

The training, validation, and test sets were partitioned at a ratio of 70:15:15 by user ID to prevent data leakage across sets. For the style learning and virtual try-on modules, we further ensured category-wise stratification to maintain proportional representation of garment types (e.g., tops, dresses, outerwear) in each split. Given the inherent imbalance in user interaction frequency and body type distribution, we applied class-aware sampling during training: samples from underrepresented body shape clusters (e.g., plus-size or petite categories) and low-frequency fashion styles were oversampled using SMOTE-inspired augmentation in the feature space. This balancing strategy improved model fairness and generalization without distorting the natural distribution in the evaluation sets.

The dataset used in the experiment was specially assembled to include all aspects of clothing modifications. It included multi-source datasets such as proprietary fashion databases, fashion WIKI pages, and specific domain files of anthropometric measurements. In particular, the dataset included 50,000 exemplar garments, 10,000 body measurement images, and 5,000 user logs describing the interactions to fully capture fashion customization attempts. Comprehensive filtering steps were performed on the data including normalization, outlier exclusion, and semantic feature extraction to improve the data quality and compliance.

The evaluation methodology imposed a multi-tiered assessment structure that included both quantitative operational indicators and qualitative user experience aspects. Their principal evaluation strategy combined econometric techniques, and a broad scope performance evaluation centred on core aspects like measurement correctness, style suggestion accuracy, virtual try-on image quality, and system effectiveness. The experimental design utilized elaborate cross-validation and bootstrap methods to maximize the validity and applicability of the findings across various fashion domains and user populations.

Mathematical representation of the evaluation framework can be expressed through a comprehensive performance metric:

14 $${P_{overall}}={\omega _1} \cdot {A_{accuracy}}+{\omega _2} \cdot {P_{recommendation}}+{\omega _3} \cdot {Q_{visualization}}+{\omega _4} \cdot {E_{efficiency}}$$

Where ${P_{overall}}$ represents the comprehensive system performance, with weighted contributions from measurement accuracy, recommendation precision, visualization quality, and computational efficiency. This multidimensional approach enables a nuanced assessment that captures the complex technological capabilities of the intelligent clothing customization system.

The proposed intelligent clothing customization system is validated using multilayered advanced computational techniques and detailed performance analysis. This created a robust framework for determining the potential and limitations of intelligent fashion personalization. Such sophisticated systems alongside comprehensive methodologies provide and evaluate the systems’ technological and practical efficacy.

In addition, a detailed table illustrating the configuration of the experiment would explain the multifaceted approach one takes to systematically validate the design and measure performance. Not only does this design validate the system, but it also sets a methodological standard for subsequent investigations concerning intelligent clothing customization technologies.

### Performance evaluation

#### Performance evaluation on public benchmarks

To ensure fair and reproducible evaluation, we benchmark our system against state-of-the-art methods on three widely used public datasets, covering body measurement, fashion recommendation, and virtual try-on tasks. All baselines are implemented using their official codebases and trained under identical conditions (same hardware, optimizer, data splits). We compare our full pipeline (Ours: **CNN–ViT–GAN**) with representative CNN variants (ResNet, DenseNet, EfficientNet), as well as recent vision transformers.

**Table 7:** Body measurement accuracy on DeepFashion3D^26^(MAE in cm, ↓ lower is better).

Method	Architecture	Chest	Waist	Hip	Avg MAE
Li et al.^32^	ResNet-50	0.82	0.91	0.87	0.87 ± 0.01
Chen et al^33^.	DenseNet-121	0.76	0.84	0.81	0.8 ± 0.012
Zhang et al^34^.	EfficientNet-B4	0.71	0.78	0.75	0.75 ± 0.002
Ours (w/o ViT)	HRNet-W48	0.42	0.45	0.41	0.43 ± 0.011
Ours (full)	HRNet + ViT	0.37	0.39	0.38	0.38 ± 0.03

Tables 7 and 8 evaluate anthropometric regression on the **DeepFashion3D** dataset (10,000 3D-scanned subjects). Our HRNet-based body module significantly outperforms standard CNN backbones due to its high-resolution feature retention. The full system (with ViT-guided refinement) further reduces error by 11.6%, demonstrating that style-context awareness improves geometric estimation (e.g., by disambiguating loose vs. fitted garments).

**Table 8:** Fashion recommendation accuracy on FashionVC^28^(Top-5 accuracy %, ↑ higher is better). Top-5 accuracy is the percentage of times the correct fashion item appears in the model’s top 5 recommendations.

Method	Architecture	Visual-only	+TEXT	+CONTEXT	Avg Acc
He & McAuley^35^	AlexNet	62.1	65.3	66.0	64.5
Wang et al^36^.	ResNet-101	71.4	74.2	75.1	73.6
Liu et al^37^.	ViT-Base	76.8	79.5	80.2	78.8
Ours (CNN-only)	ResNet-50 + CF	78.3	80.1	81.0	79.8
Ours (full)	CNN + ViT	82.6	85.0	87.4	85.0

Table 9 reports results on FashionVC, a large-scale dataset with 50 K outfits, user clicks, and contextual tags (occasion, season). Our full model achieves 87.4% accuracy when all modalities are used—surpassing pure CNN and pure ViT baselines. The gain stems from our multimodal fusion controller, which dynamically weights body constraints (e.g., “waist size < 70 cm”) and contextual rules (e.g., “formal attire for weddings”).

**Table 9:** Virtual Try-On quality on VITON-HD^29^(↑ higher is better).

Method	Architecture	SSIM	FID	LPIPS	User Preference
VITON^29^	U-Net	0.78	28.5	0.21	58.2
ACGPN^30^	ResNet + GAN	0.83	22.1	0.17	67.5
StyleAvatar^31^	StyleGAN2	0.87	18.3	0.14	74.1
Ours (w/o GAN)	SPADE (no cond)	0.85	20.4	0.16	70.3
Ours (full)	SPADE + Z_fused	0.91	15.2	0.11	82.7

Table 9 evaluates photorealism and structural fidelity on VITON-HD (14 K high-res image pairs). Our conditional SPADE generator, guided by the fused vector${{\mathbf{Z}}_{{\mathrm{fused}}}}$, achieves the best scores across all metrics. The 6.3% user preference gain over StyleAvatar confirms that body-aware conditioning reduces common artifacts (e.g., misaligned seams, unnatural draping).

CNN variants (ResNet, DenseNet, etc.) perform well on local feature tasks but lack global reasoning. ViT improves recommendation but struggles with geometric precision without CNN. Our hybrid CNN–ViT–GAN pipeline consistently outperforms all baselines by integrating local structure, global semantics, and physical constraints in a unified framework. The largest gains appear in real-world applicability (user preference, contextual accuracy), not just synthetic metrics.

The performance review of the intelligent clothing custom personalisation system is an evaluation of its technological prowess, offering deep analyses of operational informativeness, precision, and computational features. The assessment model evaluated in detail many performance aspects and provided an understanding of practical usefulness and technological sophistication.

User System Efficiency emerged as a critical issue and the most important aspect was response time. The system under consideration showed excellent and consistent computational response times with sub one second interaction latencies for all user situations. Detailed time series analysis showed that the average response time for the extraction of body measurements was 350 ms while style recommendation generation measured in at 250 ms. These system performance parameters outclass the industrial norm for recommendation customization systems and demonstrate the performance of a modern computational architecture.

Accuracy of evaluation and assessment recommendations served as another dimensional measurement of assessment. The system’s method of extracting anthropometric measurements was outstanding, as the mean absolute error margins for different body types and imaging conditions were less than half a centimeter. Remarkable performance was also demonstrated in style recommendation accuracy with over 85% accuracy in multiple user profile categories. The calculation of suggestion accuracy is given in the form of total performance measure:

15 $${A_{recommendation}}=\frac{{\sum\limits_{{i=1}}^{n} {{\text{Matched Recommendation}}{{\mathrm{s}}_i}} }}{{{\text{Total Recommendations}}}} \times 100\%$$

The analysis of resource use showed the optimal efficiency of the design’s computation functionalities. The suggested architecture was proficient in optimizing resources as it operated with low computational overhead while providing high-performance personalization features. The processor use was less than 60% throughout most of the intensive processing tasks, and memory was effectively used with the incorporated caching and model compression techniques.

Contrasting pragmatic performance with intelligent DIY clothing customization platforms proved the technological leaps in the recommended system. The Evaluation framework not only measured the performance indicators, but also revealed the capability granules, thus forming a strong foundation for future technologies in intelligent fashion personalization.

The thorough evaluation of performance gives credible proof of the system’s expected impact on clothing customization in terms of precision, efficiency, and experience with the use of systems and technologies for machine learning and computing.

### User experience evaluation

As shown in Table 10, the analyses along with the user experience evaluation form an integral part of the intelligent clothing personalized customization system validation through a system-centric and user-centric approach by assessing the effectiveness and design of the system.

**Table 10:** Comprehensive user experience metrics for intelligent clothing customization System.

Evaluation Category	Mean Satisfaction Score	Standard Deviation	Interpretation
Interface Usability	4.65	0.35	Extremely High User Satisfaction
Recommendation Accuracy	4.42	0.41	Very Good Precision in Personalized Suggestions
Virtual Try-On Experience	4.58	0.38	Highly Realistic Visualization Capabilities
Overall Satisfaction	4.55	0.37	Comprehensive System Performance Meets User Expectations

The user experience evaluation section in Table 7 shows the overall assessment of the intelligent clothing customization system in terms of the four most critical components. The table illustrates the mean satisfaction scores and the standard deviations, so that the set of values can be better understood.

The table captures the user experience throughout four main metrics systematically. The user experience in terms of satisfaction scores, ranging from 4.42 to 4.65, indicates overall satisfaction among the users in all aspects. The high standard deviations from the mean, 0.35 to 0.41, indicate that users had similar experiences.

The most notable observations from the table include the interface usability leading with the highest mean score of 4.65 and lowest standard deviation of 0.35, indicating an exceptionally intuitive and universally appreciated user interface. Recommendation accuracy shows the most variability, with a slightly lower mean score of 4.42 and highest standard deviation of 0.41, suggesting room for potential algorithmic refinement. The virtual try-on experience demonstrates strong performance with a 4.58 mean score, validating the system’s advanced visualization technologies. Overall satisfaction confirms the system’s comprehensive success, with a robust 4.55 mean score across all evaluated dimensions.

The mathematical validation of these metrics can be expressed through the comprehensive user satisfaction metric:

16 $${U_s}=\frac{1}{n}\sum\limits_{{i=1}}^{n} {({w_i} \cdot {S_i})}$$

Where ${U_s}$ represents the overall user satisfaction, *n* is the number of evaluation categories, ${w_i}$ are the weighted importance factors, and ${S_i}$ are the individual satisfaction scores.

The design of the user satisfaction survey was geared towards achieving a specific aim using a five-point Likert scale. It featured 250 respondents with different ages, genders, and engagement levels with fashion. The survey instrument was crafted to capture interface usability, recommendation accuracy, virtual try-on systems, and overall system satisfaction.

Analysis through thematic appraisal of responses presented wide qualitative findings. Respondents appreciated the personalized approach to clothing customization and noted that the system has the power to change normal clothing fashion activities. Many users noted how simple the interface is and the powerful graphics that made them feel like they were virtually shopping.

The detailed evaluation strategy offered more than just standard performance measures; it provided an all-inclusive evaluation for the intelligent clothing customization system. The condensed mean score of 4.5 out of 5 indicates that users’ expectations were not only met but surpassed within the various interactions and functionalities of the system.

As much as the results were positive, the research states there are other areas that still need improvement, such as further development of the recommendation algorithms, style options, virtual try-on features and overall user experience. This study is one of the firsts on analyzing user perceptions and technological functionalities in intelligent clothing customization, and as a result, lays down important findings for subsequent work in this constantly changing area.

The user experience evaluation ultimately substantiates the proposed system’s capability to deliver a sophisticated, user-centric approach to garment personalization. By harmonizing advanced technological capabilities with intuitive user interaction paradigms, the research demonstrates the transformative potential of intelligent clothing customization technologies in addressing contemporary fashion personalization challenges.

### Comparative analysis

The comprehensive evaluation of the intelligent clothing personalized customization system necessitates benchmarking against contemporary solutions. Table 11 presents a quantitative comparison with four state-of-the-art systems across critical performance dimensions.

**Table 11:** Comparative analysis of intelligent clothing customization Systems.

Performance Metric	Proposed System	Interactive Design System	Ethnic Customization Platform	Metaverse-Based Framework	Anthropometric ML System
Measurement Precision (MAE, cm)	0.38	0.62	0.51	N/A	0.75
Style Matching Accuracy (%)	87.4	79.2	76.4	82.5	71.8
Visualization Fidelity (SSIM)	0.91	0.83	0.88	0.92	0.76
Response Time (ms)	285	425	370	620	310
Computational Efficiency (FLOPs ×10⁹)	4.3	7.8	5.2	12.4	3.6

Our system demonstrates superior measurement precision with 38.7% lower mean absolute error compared to the Interactive Design System^4^(*p* < 0.01, paired t-test), while achieving 10.4% higher style matching accuracy (*p* < 0.05). The proposed architecture surpasses previous implementations in operational efficiency, exhibiting a 32.9% reduction in response time compared to the industry average (statistically significant across all comparisons with *p* < 0.01), while maintaining visualization fidelity comparable to the Metaverse-Based Framework approach^12^(*p* = 0.68, indicating no statistically significant difference). These statistical significance tests were conducted across multiple evaluation runs (*n* = 5) using identical test datasets, confirming that the performance improvements are robust and not attributable to random variation or sampling bias.The principal advantages of the proposed system derive from: (1) the hybrid neural architecture that balances feature extraction depth with computational efficiency; (2) the implementation of knowledge distillation techniques that reduce model complexity while preserving predictive power; and (3) the multimodal data fusion approach that enhances prediction robustness across diverse usage scenarios.

User satisfaction was evaluated through two complementary studies:

- A controlled single-blind user study involving 120 participants (62 female, 55 male, 3 non-binary; age: 18–55, mean = 32.4), recruited via university and online fashion communities, with diverse body types (BMI 18.5–34.2) and fashion engagement levels. Participants rated the system on a 5-point Likert scale, yielding scores between 4.42 and 4.65 across three dimensions: recommendation accuracy, interface usability, and visualization quality. All participants received a $15 e-voucher as compensation.
- The ethical approval for this study was granted by the Academic Ethics Review Committee of the University of Southampton. All procedures involving human participants were conducted in accordance with the Declaration of Helsinki (2013 revision) and all relevant national and institutional guidelines and regulations for sports science research involving human subjects, and informed consent was obtained prior to participation.
- Additionally, a large-scale deployment test with 250 users in a simulated e-commerce environment reported an average overall satisfaction score of 4.55/5.0, consistent with the controlled study findings.

Despite these advancements, several limitations warrant acknowledgment. The current implementation exhibits reduced performance for non-standard body morphologies where training data representation is insufficient. Additionally, the system’s visualization capabilities, while advanced, cannot fully replicate physical fabric properties such as tactile sensation and micro-draping behavior. Furthermore, the computational architecture presents deployment challenges in resource-constrained environments, particularly mobile platforms without dedicated neural processing units.

These comparative insights illuminate both the significant advancements achieved and the remaining challenges that define future research trajectories for intelligent clothing personalization systems.

## Conclusion and future work

This research has successfully developed an intelligent clothing personalized customization system integrating deep learning with computer vision and style preference learning methodologies. The system achieved superior performance metrics (0.38 cm mean absolute error in anthropometric measurements, 87.4% style matching accuracy, 285ms response time), significantly outperforming existing solutions. The primary contributions include a novel framework for multimodal data fusion dynamically weighting visual, textual, and behavioral signals, and a mathematical formulation for style similarity incorporating semantic and aesthetic dimensions. With the microservices architecture, flexibility of implementation is enhanced, and it is possible to reduce measurement errors by 38.57% while decreasing return rates through more accurate garment fitting.

While achieving these goals, the research purposefully identifies some gaps. Some skeletons with non-standard body morphologies have reduced performance because they have not received adequate training. The virtual try-on system is unable to simulate the intricate tactile properties of textiles, and the computational resources requisite to do so are not always available in low-resource environments. The assessment was mostly done for casual and formal garments, which does not cover specialized clothing and does not fully represent geographic and cultural style differences.

Social and ethical issues must be kept in mind, especially in relation to the collection of anthropometric data. Even though the system employs privacy protection measures such as anonymization, encryption, and user consent, there remains a wider issue of how individuals view their body, demographic stereotypes, and the possible perpetuation of negative fashion standards. Future implementations must consider algorithmic fairness in order to mitigate algorithmic discrimination.

To expand the scope of anthropometric training data for cross-demographic augmentation, research can attempt to apply haptic feedback devices for virtual trying on, or work on edge computing for mobile deployment. First observations show a possible reduction in model size from 78% with a degradation in accuracy to 4.2%. Other work includes the development of adaptive fabric sensors for automatic changes in fit and design of style recommendation systems with respect to aesthetic values of different cultures.

In conclusion, the research conducted contributes to automated customization of intelligent clothing by showing how advanced machine learning can be used to develop meaningful fashion personalization options. By merging complexity of technology with intricate user preference, the system is capable of transforming garment personalization, and in turn, can have great impact on the radically changing world of fashion.

Regarding future directions, we outline a phased implementation roadmap: (1) within 12 months, prototype haptic feedback integration using off-the-shelf vibrotactile gloves for basic texture simulation; (2) by 18 months, deploy a quantized model (target: <5 M parameters) on mobile via TensorFlow Lite for edge-based inference; and (3) within 24 months, collaborate with textile labs to incorporate low-cost stretch/pressure sensors for real-time fit adaptation in pilot smart garments.

## Data Availability

The original contributions presented in this study are included in the article/supplementary material. Further inquiries can be directed to the corresponding author(s).
